# Saccadic Eye Movements Minimize the Consequences of Motor Noise

**DOI:** 10.1371/journal.pone.0002070

**Published:** 2008-04-30

**Authors:** Robert J. van Beers

**Affiliations:** 1 Physics of Man, Helmholtz Institute, Utrecht University, Utrecht, The Netherlands; 2 Department of Neuroscience, Erasmus MC, Rotterdam, The Netherlands; University of Minnesota, United States of America

## Abstract

The durations and trajectories of our saccadic eye movements are remarkably stereotyped. We have no voluntary control over these properties but they are determined by the movement amplitude and, to a smaller extent, also by the movement direction and initial eye orientation. Here we show that the stereotyped durations and trajectories are optimal for minimizing the variability in saccade endpoints that is caused by motor noise. The optimal duration can be understood from the nature of the motor noise, which is a combination of signal-dependent noise favoring long durations, and constant noise, which prefers short durations. The different durations of horizontal vs. vertical and of centripetal vs. centrifugal saccades, and the somewhat surprising properties of saccades in oblique directions are also accurately predicted by the principle of minimizing movement variability. The simple and sensible principle of minimizing the consequences of motor noise thus explains the full stereotypy of saccadic eye movements. This suggests that saccades are so stereotyped because that is the best strategy to minimize movement errors for an open-loop motor system.

## Introduction

**Figure 1:**
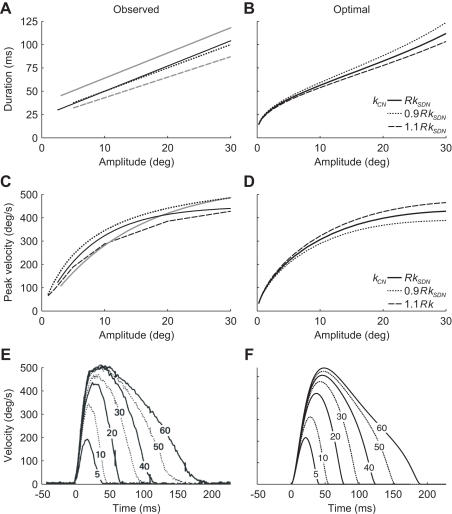
Observed and optimal main sequence. A. Observed duration as a function of amplitude for horizontal saccades starting from the primary position or moving symmetrically about it. Different lines denote linear fits reported by different sources: -––[2], -––[3], —[4], -––[5], ……[6]. Lines are plotted for the range of amplitudes for which the fit was made. B. Optimal duration as a function of amplitude for horizontal saccades moving symmetrically about the primary position for three different levels of CN (*k_CN_* = *Rk_SDN_* is the best estimate of the actual level [12]). C. Observed peak velocity as a function of amplitude for similar horizontal saccades as shown in A. Different lines denote fits or linearly connected data points reported by different sources (see legend of A for the sources). D. Peak velocity of optimal saccades as a function of amplitude for horizontal saccades moving symmetrically about the primary position for three different levels of CN. E. Observed velocity profiles of horizontal saccades moving symmetrically about the primary position for amplitudes of 5, 10, 20, 30, 40, 50 and 60 deg (Reprinted from [4] with permission from Wiley-Blackwell). F. Velocity profiles of the optimal saccades (with their optimal duration) shown in E.

We have no voluntary control over the duration and velocity of our saccadic eye movements. Normal saccades are therefore stereotyped and follow the so-called ‘main sequence’ [1]–[6]: saccade duration increases approximately linearly with saccade amplitude (Figure 1A) whereas peak velocity increases with amplitude at a decreasing rate (Figure 1C). The empirical main sequence relationships for horizontal saccades differ somewhat across studies because they vary across subjects [2], [7], measurement techniques [8], [9] and analysis method [10], but the general pattern is always the same. Although it is well understood how the stereotyped saccades are generated by the brain, it is not understood why they are so stereotyped and why they have the precise properties as described by the main sequence. The stereotyped behavior is likely to be advantageous, but in which respect are main sequence saccades advantageous?

On top of the stereotypy, saccades display a certain level of variability [6], [11], [12]. Movement variability is undesirable because it leads to failures to reach the desired gaze direction. The larger the variability, the larger the errors will be. A considerable proportion of the variability is caused by noise in the motor commands [12]. The detrimental effect of motor noise could be minimized by choosing, from the infinite number of possible saccade trajectories to a target, the trajectory that produces the smallest variability in saccade endpoints [13]. The precise properties of the motor noise determine which trajectories are optimal [14].

The noise in individual motoneurons is in a good approximation signal-dependent noise (SDN) [15]–[17], i.e., the standard deviation of the firing rate is proportional to the mean firing rate. It has been shown that, under the assumption that motor noise is SDN, the theoretical trajectories that minimize endpoint variability are very similar to actual saccade trajectories [13]. It is however not reasonable to assume that the actual motor command, which is the aggregate of the command activities of all motoneurons contributing to a saccade, has SDN because this aggregate command combines the activities of many motoneurons that are distributed over six different muscles. Especially the coactivation of antagonistic muscles [18] can lead to substantial departures from SDN because the torques generated by these muscles will partially cancel each other but the variances therein will add up. Indeed, studies aimed at identifying the properties of noise in the motor commands of saccades [12], and also of arm movements [19], found that the noise is best characterized as a combination of SDN and constant noise (CN), which is additive noise with a standard deviation independent of the command. There is also temporal variability, which leads to variations in speed and duration, but this is less relevant here because it does not lead to variations in saccade endpoints [12].

The aim of this study was to determine how actual saccades relate to the theoretical movements that minimize the consequences of the actual motor noise. The results show that they are very similar, which suggests that saccades are planned in such a way that the movement variability is minimized.

## Results

**Figure 2:**
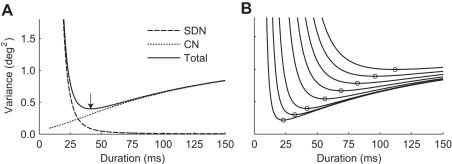
Effect of duration on endpoint variance. A. The optimal trajectory was calculated for a horizontal saccade of 5 deg (starting from the primary position), where the hypothetical movement duration was varied between 20 and 150 ms. The variance resulting from SDN and CN is plotted as a function of the duration. The total variance, which is the sum of the variances caused by SDN and CN, has a minimum value for a duration of 41.5 ms, as indicated by the arrow. This is the optimal duration. B. Total variance curves as in A for saccades with amplitudes of 1, 2.5, 5, 10, 15, 20, 25 and 30 deg (from bottom to top). The optimal durations are indicated by the circles.

We calculated (see Materials and Methods) the trajectories that minimize the endpoint variability caused by the empirically estimated combination of SDN and CN [12]. We first consider a 5 deg horizontal saccade. Although in normal behavior, the duration is given by the main sequence, we will consider a range of hypothetical durations and for each duration calculate the trajectory that produces the smallest variance in saccade endpoints, averaged over a 50 ms post-movement fixation period [13]. Figure 2A shows that the endpoint variance caused by SDN decreases with duration. This is because a saccade with a longer duration requires smaller and therefore less noisy motor commands than a saccade with a shorter duration. In contrast, the variance caused by CN increases with duration. CN is independent of the motor command, so the amount of noise added simply increases with duration. As a result, the total endpoint variance becomes very large for very short durations (because of SDN) and for very long durations (because of CN) and reaches a minimum at an intermediate duration. This means that there is an optimal saccade duration for which the endpoint variance is minimal. For the 5 deg saccade, this optimal duration is 41.5 ms, which is close to the actual duration (Figure 1A). Figure 2B shows a family of total variance curves for saccades with amplitudes of 1, 2.5, 5, 10, 15, 20, 25 and 30 deg. The optimal duration, indicated by the circles, increases with saccade amplitude.

We calculated the optimal duration for horizontal saccades of amplitudes between 0.25 and 30 deg that moved the eye symmetrically about the primary position, the eye's equilibrium position when it looks straight ahead. Figure 1B shows that the optimal duration (the bold line) increases approximately linearly with amplitude, very similar to the observed duration (Figure 1A). Note that the larger slope that is optimal for very small amplitudes has also been observed [1], [20], [21] but is generally not included in the linear amplitude-duration fits. Peak velocity of the optimal saccades increases with amplitude at a decreasing rate (Figure 1D), also very similar to the observations (Figure 1C). The velocity profiles of the optimal saccades (Figure 1F) have similar shapes as observed velocity profiles (Figure 1E). The initial part of the movement is similar for all amplitudes, and velocity profiles are approximately symmetric for small saccades, and asymmetric with an extended deceleration phase for larger saccades.

The optimal duration is determined by the ratio *R* of the levels of CN and SDN, and by the mechanical properties of the oculomotor system (see Materials and Methods). Since the levels of CN and SDN are likely to vary across individuals, we calculated the optimal duration for noise ratios *R* that were 10% larger and smaller than the best estimate [12]. Figure 1B shows that the optimal duration decreases when there is relatively more CN and it increases with less CN, but both curves fall within the range of observed durations (Figure 1A). The optimal peak velocity (Figure 1D) shows a corresponding dependence on the noise ratio. These variations also fall within the range of observed peak velocities (Figure 1C).

The mechanical properties of the oculomotor system are less likely to vary much between humans but they differ strongly across different species [22], [23]. The largest time constant of the monkey oculomotor system (modeled as a linear system), for instance, is about half that of the human system [24]. As a result, the optimal duration is considerably shorter for monkeys than for humans (when assuming the same noise ratio *R* as for humans). Monkey saccades are indeed about a factor two faster than human saccades [25]. Conversely, the oculomotor systems of the cat [23] and rabbit [22], [23] are characterized by longer time constants and their saccades are slower than human saccades [26], [27].

**Figure 3:**
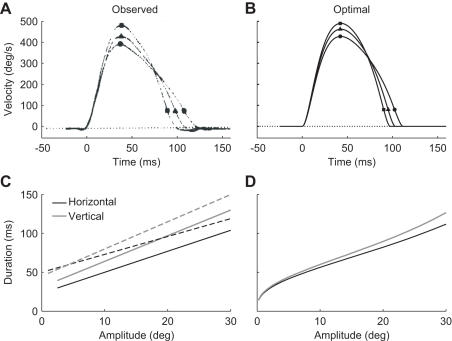
Effect of movement direction on saccade velocity and duration. A. Observed velocity profiles of three 30 deg rightward saccades, starting at –▪– 20, –▴–10 and –•– 0 deg to the left of the primary position (Reprinted from [28] with permission from Elsevier). B. Velocity profiles of the optimal saccades for the situation shown in A. C. Linear fits of observed duration as a function of amplitude for horizontal and vertical saccades. The solid lines are from [4] and [29], and the dashed lines are from [30]. D. Optimal duration as a function of amplitude for horizontal and vertical saccades moving symmetrically about the primary position.

The duration of a human saccade is not determined by its amplitude only, but it depends also on the movement direction and the initial eye orientation. Centrifugal saccades, which move the eye from the primary position to an eccentric position, have longer durations, lower peak velocities and more asymmetric velocity profiles than centripetal saccades, which return the eye from an eccentric position to the primary position [4], [28] (Figure 3A). This is exactly what is optimal for minimizing the consequences of motor noise (Figure 3B). The explanation is that the elastic forces of the eye counteract centrifugal saccades whereas they assist centripetal saccades. As a result, centripetal saccades require smaller torques, have less SDN and a shorter optimal duration than centrifugal saccades.

Saccades in the vertical direction have a longer duration than horizontal saccades of the same amplitude [4], [29], [30] (Figure 3C). This is also optimal for minimizing the consequences of motor noise (Figure 3D). A comparison of the predicted and observed duration differences is however difficult because the observed differences vary largely across studies (see Figure 3C). The optimal durations are different for horizontal and vertical saccades because the horizontal extraocular muscles are stronger than the vertical ones. Stronger muscles are less noisy than weaker muscles when both produce the same torque [31]. The levels of SDN and CN are therefore lower in the horizontal than in the vertical muscles. SDN is only present for muscles that are activated. As a result, there will be less SDN for horizontal than for vertical saccades. In contrast, the level of CN is independent of the motor commands, so there will always be CN in all muscles, even in vertical muscles during a horizontal saccade, and vice versa. In other words, the level of CN will for each muscle pair always be the same. Summed over all muscle pairs, there will be less SDN for horizontal than for vertical saccades but the amount of CN will be the same. This leads to a shorter optimal duration for horizontal saccades.

What is the duration of saccades in oblique directions? Let us assume that a 10 deg saccade to the right takes 56 ms and an upward one 60 ms. How long does an oblique saccade 10 deg to the right and 10 deg up then take? Since the oblique saccade requires similar horizontal muscle activations as the rightward saccade and comparable vertical muscle activations as the upward saccade, one could expect its duration to be in the range of that of the rightward and upward 10 deg saccades, i.e., 56–60 ms. That would mean that oblique saccades would be ‘superfast’ because they would be faster than horizontal and vertical saccades of the same amplitude (about 14 deg, taking 67–71 ms). Actual oblique saccades are however not superfast, but their duration compares to that of purely horizontal and vertical saccades of the same amplitude [12], [32]. Why is the duration not shorter? Although the muscles could in principle generate such a superfast saccade, the endpoint variability would be larger than necessary. For the optimal duration, the balance between SDN and CN, summed over all muscle pairs, is the same as for purely horizontal and vertical saccades. A shorter duration would lead to an imbalance because there would be too much SDN.

**Figure 4:**
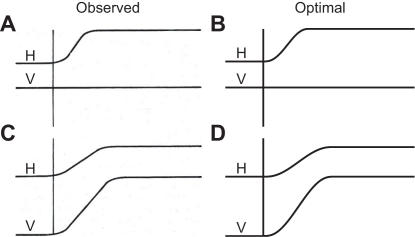
Component stretching in oblique saccades. A. Observed time course of a 5 deg purely horizontal saccade (H and V denote the horizontal and vertical components, respectively) (Reprinted from [32] with permission from APS). B. Optimal time course of a 5 deg purely horizontal saccade. C. Observed time course of an oblique saccade with a 5 deg horizontal component and a 10 deg vertical component (Reprinted from [32] with permission from APS). D. Optimal time course of an oblique saccade as shown in C.

Another feature of oblique saccades is that their horizontal and vertical components have approximately the same duration, even when their amplitudes are different [32], [33] (Figure 4C). The peak velocity of each component is therefore lower and the duration longer than for a saccade for which the considered component has the same amplitude but a zero orthogonal component (Figure 4A). This ‘component stretching’ is optimal for minimizing the consequences of motor noise (Figures 4B,D). Unequal durations would lead to strongly curved trajectories, which would require a larger total rotation angle and therefore larger and noisier motor commands, with a larger variability as the result. The optimal trajectories are therefore straight, with the same duration for the horizontal and vertical components.

**Figure 5:**
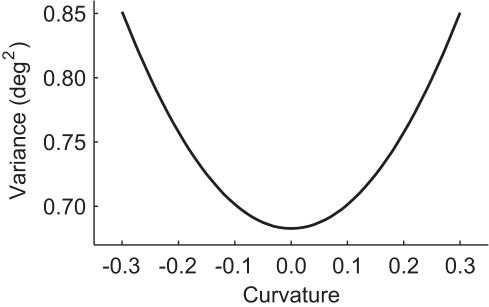
Endpoint variance as a function of trajectory curvature. Oblique 15 deg saccades directed up and to the right (under 45 deg) are considered for a range of trajectory curvatures. Curvature was expressed as the ratio of the perpendicular deviation from the straight line between saccade onset and offset and the net amplitude of the saccade, with positive values indicating detours in the anti-clockwise direction and negative values detours in the clockwise direction. The curved paths were assumed to follow a sinusoidal shape in rotation vector space. The variance is minimal for straight trajectories and increases with curvature.

The trajectories of actual oblique saccades can display some curvature [34], [35]. The amount and even the direction of this curvature vary strongly across and sometimes also within subjects [34], [35]. The curvature, when expressed as the ratio of the perpendicular deviation from the straight line between saccade onset and offset and the net amplitude of the saccade, is generally 0.1 or smaller [34]. We performed an additional analysis to find the relation between saccade curvature and endpoint variance. Figure 5 shows the endpoint variance resulting from motor noise for a 15 deg saccade up and to the right as a function of curvature. The figure confirms that the variance is minimal for zero curvature, and that it increases with increasing absolute curvature. The increase is marginal (less than 3%) for absolute curvatures up to 0.1. The variance increases more rapidly for larger curvatures. For a curvature of 0.2 there is a 10% increase and for a curvature of 0.3 the increase is almost 25%.

## Discussion

We make saccadic eye movements to direct our fovea quickly to objects of interest. Saccades thus serve vision. It can therefore be expected that the saccadic system has evolved in such a way that it serves vision optimally. It is however not obvious what saccades should look like to support vision optimally. Saccade duration seems important for two reasons. First, when we detect an object of interest, such as a possible predator, in our visual periphery, it is important to direct our gaze to that object as fast as possible. Second, because vision is highly degraded during a saccade, vision is served best by making the duration of a saccade as short as possible. It could therefore be expected that saccades have evolved to be as fast as possible [36]–[38]. The duration of saccades in oblique directions, however, argues strongly against this possibility. If saccade duration were minimized, oblique saccades would be ‘superfast’, but they clearly are not [12], [32].

Another important feature of saccades is their accuracy. Individual saccades can miss the desired destination as a result of motor noise and uncertainty in the sensed target location. Such errors can have devastating consequences. For instance, a saccade error may preclude the timely identification of a predator (or prey). Our survival may therefore depend on saccade accuracy. This demonstrates that minimizing variability in saccade endpoints is behaviorally relevant because the smaller the variability, the smaller the mean error will be. The relevance goes however further than this. Once an error has been made, a secondary, corrective saccade will be generated. The number and the amplitude of the required secondary saccades, and therefore the average time needed to reach the desired destination, will all be close to minimal when the endpoint variance is minimized. Minimizing the endpoint variance thus indirectly also approximately minimizes the total time with impaired vision during saccades and the time needed to reach the desired gaze direction.

Our results show that the full stereotypy of saccade trajectories and durations is optimal for minimizing the variability in saccade endpoints. Given the above-mentioned advantages, we propose that the saccade system has purposefully been optimized to minimize this variability. It is possible that this optimization process has taken place during evolution, but another possibility is that the optimization has occurred during the development of each individual. Some support for the latter option comes from the observation that saccade duration varies somewhat across subjects [2], [7]. This could be related to inter-individual differences in the noise levels, but more research is required to test whether this relation really exists. The finding that the sign and magnitude of the curvature of oblique saccades vary across subjects [34], [35] provides stronger support for the latter option. Figure 5 shows that for the curvatures that oblique saccades typically have (absolute value not greater than 0.1), the endpoint variance is only marginally larger than that of the optimal straight saccades. For larger curvatures, the variance can be substantially larger. A plausible mechanism therefore is that the saccade optimization process is driven by the errors that are experienced after making saccades. If at a certain time during this optimization process highly curved saccades are produced, the central nervous system could sense that the saccade errors tend to be larger than when less curved trajectories are made. This will induce a change towards planning of less curved saccades. This process will continue until no further improvements can be made. At absolute curvatures of 0.1, the improvement that could still be made can be too small (less than 3% reduction of variance) to be detectable. In that case, reducing the curvature will stop and the optimization process has found a solution. This solution differs somewhat from the theoretical optimum, but the resulting variance is hardly larger. The fact that curvature can have opposite signs in different subjects is consistent with this mechanism because the optimization process will follow a different path in every individual, due to the different realizations of motor and sensory noise in the saccades made during the optimization process.

Which trajectories and durations are optimal depends on the precise properties of the motor noise [14]. Previous studies [13], [38], [39] assumed that motor noise is pure SDN. Later work [12], [19] has shown that this assumption is not correct. The present work can be seen as an extension of the seminal work of Harris and Wolpert [13]. In comparison to their study we have replaced the assumption that motor noise is pure SDN by the empirically established actual motor noise. The result is that we can explain much more: not only the velocity profiles (as Harris and Wolpert did) but the full stereotypy of saccade trajectories including the duration of saccades, and how duration and velocity vary with movement direction and initial eye orientation.

In addition to explaining what the stereotypy looks like, the principle of minimizing the consequences of motor noise also explains why there is a stereotypy in the first place. Without the stereotypy, duration and velocity would be more variable. Many saccades would therefore be suboptimal and produce large errors. The only way to avoid this is to always choose a trajectory that is close to the optimal trajectory, or, in other words, to produce stereotyped saccades. This may seem a very general principle that must apply to the goal-directed movements of other body parts as well. The movements of many other body parts are however less stereotyped. We have, for instance, voluntary control over the duration and velocity of arm movements. Why are arm movements less stereotyped? This could be related to their longer duration, which makes it possible to correct movements online on the basis of sensory feedback [40]. Online corrections can prevent movement errors without the need to compute an entire, optimal trajectory in advance. Stereotyped movements are therefore only optimal for open-loop motor systems such as the saccade system for which movements cannot be corrected online.

Although we have shown that a single, simple principle can explain the full stereotypy of saccade durations and velocity profiles, there is one aspect of saccade trajectories that we did not consider. The torsion of the eye was assumed to obey Listing's law. In principle, however, torsion is not constrained to obey Listing's law but it is also a free parameter. It is unknown why the actual eye orientation does obey Listing's law [41]. Based on the present work, an attractive hypothesis would be that also Listing's law minimizes the consequences of motor noise. Future research is required to test this hypothesis.

In summary, we have shown that the stereotyped durations and velocities of saccadic eye movements are optimal for minimizing the variability in saccade endpoints caused by motor noise. This suggests that the saccade system has purposefully been optimized to minimize the consequences of motor noise. This optimization process could have taken place during evolution, but it is more likely that it takes place during the development of each individual. A key element of the study is that we minimized the consequences of the recently estimated actual motor noise, rather than that we, incorrectly, assumed signal-dependent noise. This study therefore stresses that, in studies in which motor noise plays a role, it is very important to make correct assumptions about the properties of this noise.

## Materials and Methods

### Mechanics of the oculomotor system

We used the same three-dimensional model of the oculomotor system that we used to estimate the properties of motor noise [12]. In brief, the muscle torques must counteract inertial, viscous and elastic forces [42], [43]:
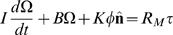
(1)where the eye orientation is described by a rotation of angle φ about axis **n̂** from the primary position; Ω and 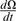 denote the eye's angular velocity and angular acceleration, respectively. The moment of inertia was *I* = 2.00×10^−7^ kg·m^2^ [44]. The coefficient of viscosity *B*, and the stiffness *K* were chosen such that (1) corresponds to an overdamped system with time constants of 224 and 13 ms [45].

The right hand side of (1) represents the torque generated by the extraocular muscles. These muscles were assumed to form three pairs with orthogonal insertions on the globe. The net torques generated by these muscle pairs form the vector **τ**. This vector is multiplied by matrix *R_M_*, that describes a rotation of angle 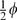 about axis **n̂** [46], to accommodate the effects of muscle pulleys [47] and/or orbital fat [48] on the muscle pulling directions.

The muscles produce torques because they receive motor commands from their motoneurons. We modeled the muscles as first order lowpass filters with a time constant of *t_m_* = 10 ms [12], [13], [39] to define the relation between the torques **τ** and the aggregate motor command 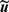: 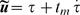, where 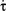 is the temporal derivative of **τ**. We next expressed eye orientation as three-dimensional rotation vectors **r** [49]. The three elements represent the torsional, vertical and horizontal components, respectively. After making this transformation and including the muscle model, equation of motion (1) is very well approximated by (errors <0.2% for normal saccades):
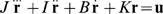
(2)where **r** ˙, 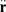 and 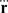 are the first, second and third temporal derivatives of **r**, respectively, and *J* is a constant. Equation (2) describes a three-dimensional, linear, overdamped system with time constants *t*_1_ = 224 ms, *t*_2_ = 13 ms and *t*_3_ = 10 ms, and with input 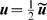 (the factor 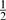 arises from the transformation to rotation vectors).

When we define the state vector as: 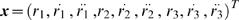, (2) can be written in the form of the state equation:
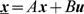
(3)where *A* and *B* are:
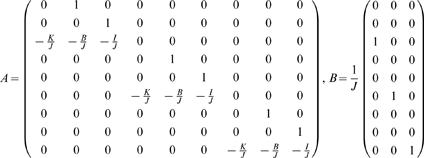
(4)The solution of (3) is [50]:
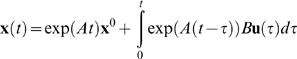
(5)


### Motor noise

We assumed that motor commands have constant noise (CN) and signal-dependent noise (SDN) in their magnitude [12]. CN and SDN are zero-mean, white Gaussian noise, with standard deviations *k_CN_* and 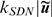, respectively. For the horizontal muscles, *k_CN_* = 1.37·10^−5^ *kg* *m* ^2^ *s* ^−2^ and *k_SDN_* = 0.172 [12]. Noise levels vary across muscle pairs as torsional∶vertical∶horizontal = *n*_1_∶*n*_2_∶*n*_3_ = 1.41∶1.41∶1.00 to reflect the different muscle strengths [12], [31].

### Optimal trajectories

We calculated optimal saccade trajectories that, on average, bring the eye to the target with minimal variance in eye orientation, summed over the horizontal, vertical and torsional components, and averaged over a post-movement fixation interval *F* [13].

The shape of a saccade trajectory is fully determined by the shape of the motor command. Motor commands of saccades are generally assumed to consist of two parts. The pulse component consists of large signals that set the eye into motion and bring it to its end position. The smaller signals of the step component are then required to keep the eye stationary after the movement. We define the duration *M* of a saccade as the duration of its pulse component. Let *t_F_* be the time in the interval [0, *F*], then the variance in the fixation interval [*M*, *M*+*F*] in component *i* resulting from SDN is [51]:
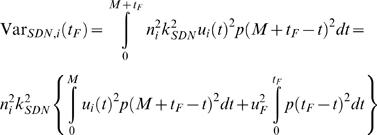
(6)where *u_F_* is the fixation command required to keep the eye still at the target position, and *p*(*t*) is the impulse response function which for all three components is:
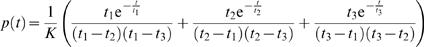
(7)Similarly, the variance in component *i* resulting from CN is:
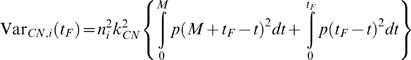
(8)The cost to be minimized is the total variance summed over the three components and averaged over the fixation interval:
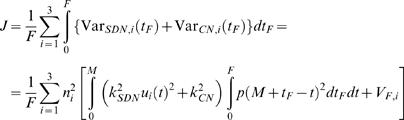
(9)with: 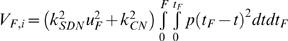, which is independent of the trajectory and the duration so that it can be omitted from the cost function. This means that the optimal trajectory and duration are independent of the noise in the step component of the motor command. The constant 1/*F* is also irrelevant for the cost function, so the cost can be simplified to:
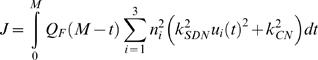
(10)with: 
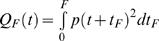
.

The optimization problem is further specified by the constraints that the eye moves from initial state **x** ^0^ at *t* = 0 to final state **x** *^f^* at *t* = *M*. Substituting the initial and final states in (5) defines the nine constraints as:
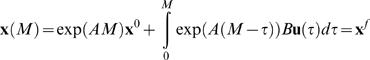
(11)The optimization problem can thus be formulated as:
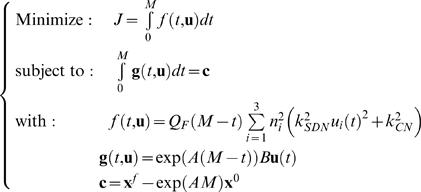
(12)The optimal motor commands and the resulting optimal trajectories can be found using calculus of variations. Let *H* = *f*+Γ*^T^* **g** be the augmented cost function, where Γ = (γ_1_,…,γ_9_)*^T^* is a vector of nine Lagrange multipliers γ*_i_*. Then, the solution of the optimization problem can be found by solving the Euler-Lagrange equation [52]:
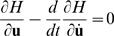
(13)The solution for the optimal motor command, for a given *M*, is:
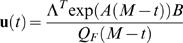
(14)where **Λ** is a nine dimensional vector of constants λ*_i_* that are determined by the nine constraint equations. This solution is identical to the solution if there were no CN. This means that, for a given *M*, the optimal trajectory in the presence of both SDN and CN is identical to the optimal trajectory in the presence of SDN only, and that was found previously [13].

The solution for the λ*_i_* can be shown to be: 
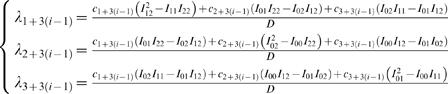
 for *i* = 1, 2, 3 and with: *D* = *I*_00_(*I*_12_^2^−*I*_11_ *I*_22_)+*I*_01_(*I*_01_ *I*_22_−2*I*_02_ *I*_12_)+*I*_02_^2^ *I*_11_, and: 
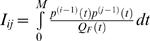
, where *p* ^(*i*)^(*t*) is the *i*th temporal derivative of *p*(*t*).

The trajectories of the optimal movements can be found by substituting (14) into (5).

### Optimal duration

To find the optimal duration *M*, we consider *M* as a free endpoint and apply the transversality condition [52]:
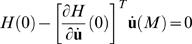
(15)Solving (15) leads eventually to the equation:
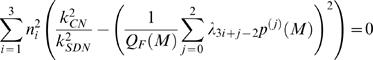
(16)This is an implicit equation for the optimal *M* that has a single solution. The equation shows that the optimal duration depends on the ratio *R* = *k_CN_*/*k_SDN_* of the levels of CN and SDN, not on these levels themselves. The optimal duration depends also on the mechanics of the oculomotor system (via the impulse response function), on the fixation period *F* (via *Q_F_*(*M*)) and, of course, on the initial and final eye orientation (which determine the λ*_i_*).

### Simulations

We assumed that the eye orientations at movement onset and offset obeyed Listing's law. This means that the first elements of **x** ^0^ and **x** *^f^* were zero. As a result, the full optimal trajectories appeared to obey Listing's law. We also assumed that the velocity and acceleration at movement onset and offset were zero (i.e., the elements 2, 3, 5, 6, 8 and 9 of **x** ^0^ and **x** *^f^* were zero).

Although we derived expressions for the optimal trajectories, these trajectories could not be calculated in closed form because the integrals *I_ij_* cannot be solved analytically. These integrals were solved numerically using a time-step of 0.025 ms. The optimal duration was determined by solving (16) numerically, also using a time-step of 0.025 ms. The post-movement fixation interval *F* was set to 50 ms. Making this interval longer led to negligible changes to the optimal durations and trajectories.

We assumed that motor noise is a combination of SDN and CN. Actual saccades, however, also display temporal variations, which are simultaneous variations in duration and velocity across repeated movements, such that the saccade amplitude is constant [12]. The actual durations are approximately normally distributed with a standard deviation of 0.068 times the mean duration [12]. Although this temporal variability does not directly induce variability in saccade endpoints, it is formally not correct to assume that the duration is constant when determining the optimal durations and trajectories. However, estimates showed that the effects of the temporal variability on the optimal duration and trajectory are negligible. The effect on the optimal duration, for instance, is generally smaller than 0.5 ms, which is very small compared to the effect of the uncertainty in the noise levels (Fig. 1B).
