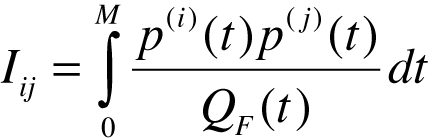# Correction: Saccadic Eye Movements Minimize the Consequences of Motor Noise

**DOI:** 10.1371/annotation/5a332f5e-aea5-40e3-8601-58bcd141b491

**Published:** 2010-07-29

**Authors:** Robert J. van Beers

In Materials and Methods, under subheading Optimal trajectories, below Equation 14, the equation for Iij is incorrect. Please view the correct equation here: